# Novel phenotype associated with a mutation in the *KCNA1*(Kv1.1) gene

**DOI:** 10.3389/fphys.2014.00525

**Published:** 2015-01-15

**Authors:** Maria C. D'Adamo, Constanze Gallenmüller, Ilenio Servettini, Elisabeth Hartl, Stephen J. Tucker, Larissa Arning, Saskia Biskup, Alessandro Grottesi, Luca Guglielmi, Paola Imbrici, Pia Bernasconi, Giuseppe Di Giovanni, Fabio Franciolini, Luigi Catacuzzeno, Mauro Pessia, Thomas Klopstock

**Affiliations:** ^1^Section of Physiology and Biochemistry, Department of Experimental Medicine, School of Medicine, University of PerugiaPerugia, Italy; ^2^Section of Neurophysiology and Biophysics, Istituto Euro-Mediterraneo di Scienza e TecnologiaPalermo, Italy; ^3^Department of Neurology, Friedrich-Baur-Institute, Ludwig-Maximilians-UniversityMunich, Germany; ^4^German Network for Mitochondrial Disorders (mitoNET)Ludwigshafen, Germany; ^5^DZNE - German Center for Neurodegenerative DiseasesMunich, Germany; ^6^Clarendon Laboratory, Department of Physics, University of OxfordOxford, UK; ^7^Department of Human Genetics, Ruhr-University BochumBochum, Germany; ^8^Center for Genomics and Transcriptomics (CeGaT) GmbH TübingenTübingen, Germany; ^9^Department of Supercomputing Applications and Innovation, CINECA (Consorzio Inter-Universitario per il Calcolo Automatico)Rome, Italy; ^10^Department of Pharmacy, University of BariBari, Italy; ^11^Neurology IV – Neuromuscular Diseases and Neuroimmunology Unit, Foundation IRCCS Neurological Institute “Carlo Besta”Milan, Italy; ^12^Department of Physiology and Biochemistry, Faculty of Medicine and Surgery, University of MaltaMsida, Malta; ^13^Department of Chemistry, Biology and Biotechnology, University of PerugiaPerugia, Italy; ^14^German Center for Vertigo and Balance DisordersMunich, Germany

**Keywords:** episodic ataxia type 1, hyperthermia, sleep, migraine, *Shaker* potassium channels, C185W

## Abstract

Episodic ataxia type 1 (EA1) is an autosomal dominant K^+^ channelopathy which manifests with short attacks of cerebellar ataxia and dysarthria, and may also show interictal myokymia. Episodes can be triggered by emotional or physical stress, startle response, sudden postural change or fever. Here we describe a 31-year-old man displaying markedly atypical symptoms, including long-lasting attacks of jerking muscle contractions associated with hyperthermia, severe migraine, and a relatively short-sleep phenotype. A single nucleotide change in *KCNA1* (c.555C>G) was identified that changes a highly conserved residue (p.C185W) in the first transmembrane segment of the voltage-gated K^+^ channel Kv1.1. The patient is heterozygous and the mutation was inherited from his asymptomatic mother. Next generation sequencing revealed no variations in the *CACNA1A*, *CACNB4*, *KCNC3*, *KCNJ10*, *PRRT2* or *SCN8A* genes of either the patient or mother, except for a benign variant in *SLC1A3*. Functional analysis of the p.C185W mutation in *KCNA1* demonstrated a deleterious dominant-negative phenotype where the remaining current displayed slower activation kinetics, subtle changes in voltage-dependence and faster recovery from slow inactivation. Structural modeling also predicts the C185W mutation to be functionally deleterious. This description of novel clinical features, associated with a Kv1.1 mutation highlights a possibly unrecognized relationship between K^+^ channel dysfunction, hyperthermia and migraine in EA1, and suggests that thorough assessments for these symptoms should be carefully considered for all patients affected by EA1.

## Introduction

Episodic ataxia type 1 (EA1) is an autosomal dominant K^+^ channelopathy which manifests as brief episodes of cerebellar dysfunction with paroxysmal ataxia, dysarthria, diplopia, vertigo and tremor often lasting for seconds or minutes. Some patients also show interictal myokymia. Additional ictal symptoms may comprise spastic contractions, stiffening of the body, visual disturbances, seizures and mild headache (VanDyke et al., 1975). The episodes can be triggered by emotional or physical stress, startle response, sudden postural change, and even caffeine. Fever or high temperatures occurring after a hot bath have also been reported to precipitate attacks (Eunson et al., 2000; Klein et al., 2004). The onset of symptoms is typically within the first or second decade of life and the frequency of attacks can vary from less than once per month to 30 times a day.

The disorder results from loss-of-function mutations in the *KCNA1* gene which encodes the voltage-gated K^+^ channel, *Kv1.1* (Browne et al., 1994; Adelman et al., 1995; D'Adamo et al., 1998; Imbrici et al., 2003, 2006, 2007, 2011; Cusimano et al., 2004; Pessia et al., 2012). Most individuals with EA1 have a pedigree suggestive of autosomal dominant inheritance with at least one affected family member. However, one *de novo* mutation has also been reported (Demos et al., 2009). Diagnosis is primarily based on clinical findings and molecular genetic testing of *KCNA1*, and these investigations in several affected families have broadened the clinical spectrum of EA1 to now include phenotypes with delay in motor development, choreoathetosis, cognitive dysfunction, transient postural abnormalities in infancy, shortening of the Achilles tendon in children and epileptic seizures (Zuberi et al., 1999; Demos et al., 2009; D'Adamo et al., 2012, 2013; Pessia et al., 2012). Electromyographic myokymia can be confirmed in nearly all individuals with EA1, but in some cases the myokymic activity only becomes apparent after application of regional ischemia. Mutations in *KCNA1* are also known to be associated with isolated neuromyotonia (Eunson et al., 2000; Kinali et al., 2004). Moderate muscle hypertrophy, a generalized increase in muscle tone and bilateral calf hypertrophy have also been reported for EA1 patients (VanDyke et al., 1975; Kinali et al., 2004; Demos et al., 2009). Recently, direct evidence identified the motor nerve as an important generator of myokymic activity and showed that dysfunction of juxtaparanodal *Kv1.1* channels alters Ca^2+^ homeostasis in motor axons (Brunetti et al., 2012; D'Adamo et al., 2014). In a large Brazilian family harboring a *KCNA1* mutation, low blood levels of magnesium with recurrent muscle cramps, tetanic episodes, limb muscle weakness and electromyographic myokymia were also found (Glaudemans et al., 2009). Since *Kv1.1* channels are widely expressed in the nervous system, particularly in the cerebellum, hippocampus and hypothalamus (Albrecht et al., 1995; Geiger and Jonas, 2000; Herson et al., 2003), it is therefore perhaps not so surprising that EA1 patients exhibit such a variable clinical phenotype (Graves et al., 2010).

In this study, we now describe a 31-year-old man carrying a dominant-negative, loss-of-function mutation in *KCNA1* who displays a new phenotype characterized by episodes of hyperthermia, short-sleep duration, and severe migraine.

## Materials and methods

### Genetic analysis

Patients were studied after giving informed consent and investigations were conducted in accordance with protocols approved by the institutional review boards of the Friedrich-Baur-Institute of the Ludwig-Maximilians-University, Munich, Germany. DNA was isolated from the peripheral blood by standard methods. The coding regions and exon-intron boundaries of *KCNA1* were PCR amplified using primer oligonucleotides designed with the Primer Express tools. PCR conditions are available upon request. Direct sequencing of *KCNA1* was performed using an automated Sanger dideoxy method.

### Electrophysiology

The human *Kv1.1* cDNA was subcloned into the pBF oocyte expression vector. The p.C185W mutation was introduced by site-directed mutagenesis and verified by automated sequencing. The concentration of *in vitro* transcribed cRNA was quantified by electrophoresis and ethidium bromide staining, and spectrophotometric analysis. Expression of wild-type and mutant channels in *Xenopus laevis* oocytes and two-electrode voltage-clamp recordings (TEVC) and patch-clamp recordings were performed as previously described (D'Adamo et al., 1998). Briefly, TEVC recordings were performed from oocytes at ~22°C and 1–8 days after cRNA injection by using a GeneClamp 500 amplifier (Axon) interfaced to a PC computer with an ITC-16 interface (Instrutech Corp., USA). Microelectrodes were filled with 3M KCl and had resistances of 0.1–0.5 MΩ. The extracellular solution contained (mM): NaCl 96, KCl 2, MgCl_2_ 1, CaCl_2_ 1.8, HEPES 5, pH 7.4. Recordings were filtered at 2 kHz and acquired at 5 kHz with Pulse software and analyzed with either PulseFit (HEKA, Germany) or Origin 8 program. Leak and capacitative currents were subtracted using a P/4 protocol. Kinetic parameters were evaluated from current amplitudes <10 μA using low resistance electrodes.

Patch-clamp recordings were performed with an Axopatch 200B amplifier (Axon Instruments). The pipette solution contained (mM): NaCl 120, KCl 2, HEPES 5, pH 7.4 whereas the cytoplasmic solution contained (mM): KCl 120, EGTA 1, HEPES 5, pH 7.4. Recording electrodes were pulled from borosilicate glass, coated with Sylgard and had resistances of 5–15 MΩ. The excised inside-out patch recordings were low-pass filtered at 0.5–2 kHz with an 8 pole bessel filter (Frequency Devices, MA) and acquired with a Pulse + PulseFit program (HEKA elektronik GmbH, Germany). Channel activity was analyzed with a TAC-TACfit program (Bruxton Co. WA), by inspecting all transitions and their slope conductance was assessed at different potentials from all events histograms.

*Xenopus laevis* were deeply anesthetized with an aerated solution containing 3-aminobenzoic acid ethyl ester methanesulfonate salt (5 mM) and sodium bicarbonate (60 mM), pH 7.3. Stage V–VI *Xenopus* oocytes were isolated, injected with 50 nl RNA and stored at 16°C in fresh ND96 medium containing (mM): NaCl 96, KCl 2, MgCl_2_ 1, CaCl_2_ 1.8, HEPES 5, gentamicin 50 μg/ml. *Xenopus laevis* underwent no more than two surgeries, separated by at least 3 weeks. Animal handling and electrophysiological experiments were conducted in accordance with international standards on animal care, and the regulations of the Italian Animal Welfare Act, approved by the local Veterinary Service Authority, and with the NIH Guide for the Care and Use of Laboratory Animals.

### Homology modeling

A 3D structural model of *Kv1.1* was built through comparative modeling using the software Modeler (Sali and Blundell, 1993) and the crystal structure of the *Kv1.2*/*2.1* chimera (PDB id.: 2R9R) as a template (Nishida et al., 2007). Sequence alignment of the target sequence *vs* the template was generated using ClustalX, and further refined using Muscle (Edgar, 2004). Twenty homology models were generated and scored against the minimum number of constraint violations. Among them, the five lowest energy models were selected and analyzed using Procheck (Laskowski et al., 1996). The final model was chosen according to the highest percentage of residues in the allowed region of the Ramachandran plot (N90%). The model was then immersed in a pre-equilibrated palmitoyl-oleoyl-phosphatidylcholine (POPC) lipid bilayer, and all overlapping lipid molecules (within 3 Å from any protein atoms) removed. The final model was further minimized to reduce steric hindrance with neighboring atoms using GROMACS4 and the GROMOS96 forcefield (Hess et al., 2008).

## Results

### Case report

**Figure 1:**
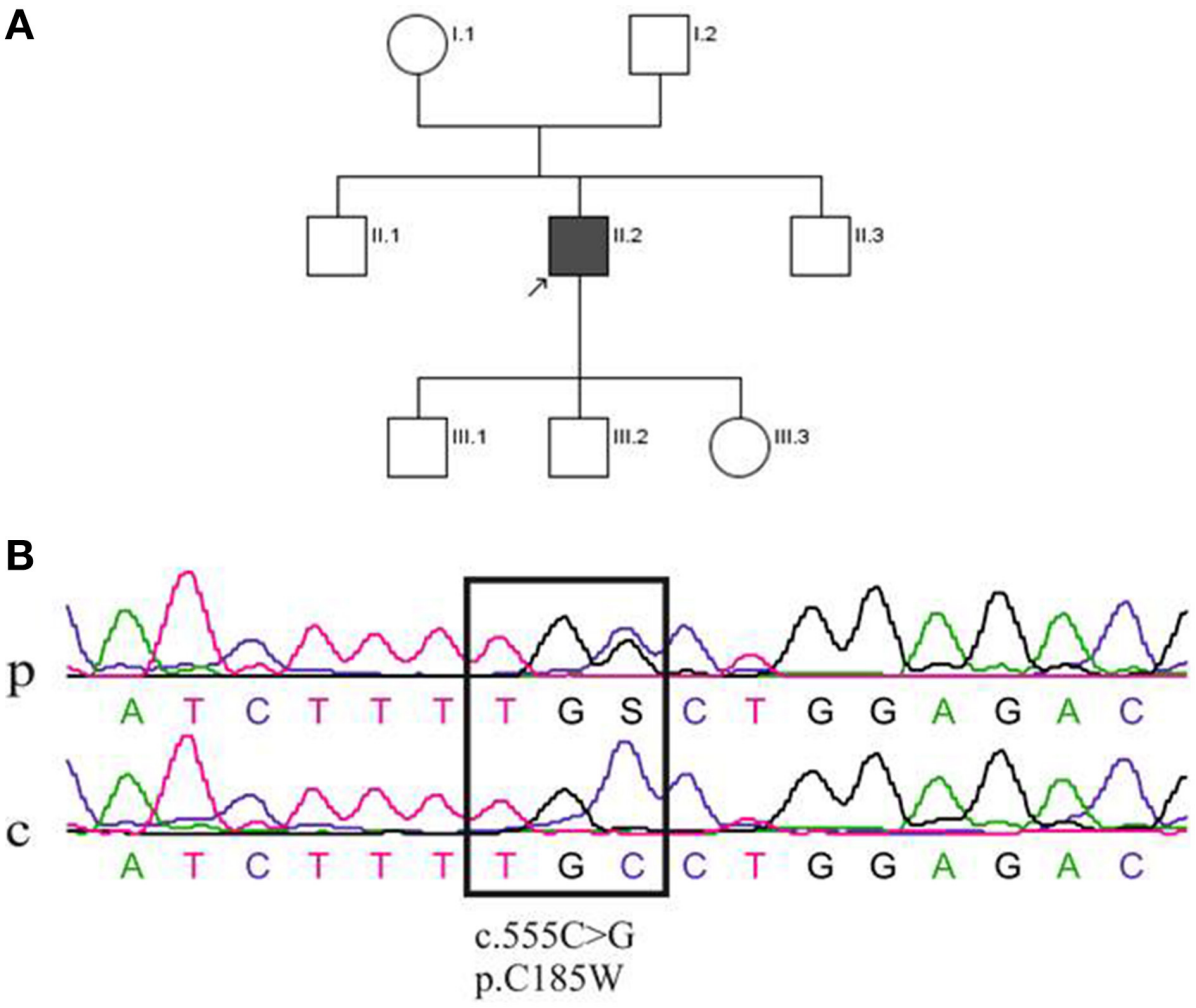
**(A)** Pedigree of the family of the patient (II.2). **(B)** Sequence analysis of the *KCNA1* gene mutation. DNA sequence from the patient (p; II2) and a control individual (c). The upper sequencing profile shows the heterozygous single nucleotide change at position c.555C > G, which leads to a cysteine (C) to tryptophan (W) substitution (p.C185W) in the protein Kv1.1.

The proband (II.2, Figure 1A) is a 31-year-old man who has been suffering from episodes of ataxia, dysarthria, diplopia and oscillopsias from childhood. The frequency of his attacks was mostly low (1–2 per week or less). Typical episodes lasted 2–3 min, were triggered by physical exercise and sudden movements, but not by alcohol or caffeine. Attacks were also precipitated by febrile illnesses. However, several episodes witnessed in-hospital were characterized by ataxia/dysarthria, lasting for hours and days, with concurrent hyperthermia up to 40.3°C that was not caused by an obvious focus of infection with the exception of streptococcal tonsillopharyngitis being detected in one episode.

With regard to his sleeping habits, the patient reported that he has always been quite active at night and had chosen to work as a night watchman. He also reported that 5–6 h of sleep were typically sufficient. Polysomnography was unremarkable (sleep stages N1, N2, N3 and REM within normal ranges, sleep latency 42 min, REM latency 56 min, apnea-hypopnea index 3, mean SaO_2_ 95.4%, min SaO_2_ 90.5% with a total sleep time being 376.6 min. No periodic leg movements or apneas were detected. The observations prompted us to investigate the sleeping habits in another EA1 patient, whose clinical case had been described previously (Imbrici et al., 2008). Interestingly, this patient also reported that just 5–6 h of sleep was sufficient although polysomnography analysis was not performed.

The proband (II.2) has suffered from severe migraine since childhood and these attacks manifested with hemicrania, nausea, photo-, and phonophobia lasting for 4–5 h. Most of migraine attacks occurred independently from ataxia, although in some instances both symptoms manifested at the same time. The clinical interictal examination of the patient was completely normal; in particular there was no myokymia, ataxia, dysarthria or oculomotor abnormalities. Electromyography without application of regional ischemia of the right biceps brachii, right abductor digiti minimi and left orbicularis oris muscles revealed no myokymia or other abnormalities. Except for a mild increase of serum osmolality, laboratory tests were normal and no abnormalities of sodium, potassium, calcium, magnesium and phosphate in blood and urine were found. Brain MRI revealed no evidence of circumscribed atrophy, and the proband was treated with acetazolamide, but this therapy had to be stopped due to tiredness. Neither of the patient's parents, aged 48 (mother, I.1) and 54 (father, I.2) years, nor his two brothers (aged 30, II.1 and 16, II.3 years), or his two sons (aged 5, III.1 and 3, III.2 years) and daughter (aged 1, III.3 year) suffered from similar episodes of ataxia or dysarthria (Figure 1A).

### Genetic analysis

Despite a negative family history, direct sequencing was performed that revealed a single nucleotide change in *KCNA1* at position c.555C > G (Figure 1B). The resulting p.C185W mutation, which changed a highly conserved residue located in the first transmembrane segment (S1) of *Kv1.1*, has been previously reported (Tomlinson et al., 2013). This heterozygous mutation was inherited from his mother, who nevertheless, appeared asymptomatic. Thus, to further associate this unusual phenotype with the c.555C > G mutation in *KCNA1*, next generation sequencing (NGS) of all known EA genes was performed. The panel comprised the following genes: *CACNA1A, CACNB4, KCNA1, KCNC3, KCNJ10, PRRT2, SCN8A*, and *SLC1A3*. This analysis confirmed the presence of the *KCNA1* c.555C > G mutation in the index patient and his asymptomatic mother. The only other sequence change found among the EA genes mentioned above was a c.1559A > G, p.K520R change in the *SLC1A3* gene of both the index patient and his asymptomatic mother. However, bioinformatics analysis suggested that p.K520R was a benign variant (PolyPhen-2: benign variant; SIFT: benign variant; Mutation Taster: pathogenic variant). These findings indicate that the c.555C > G, p.C185W mutation in *KCNA1* is likely to be causative in this patient and suggest that the asymptomatic status of his mother may be due to low penetrance of the mutation.

### Functional analysis of *Kv1.1* C185W mutation

**Figure 2:**
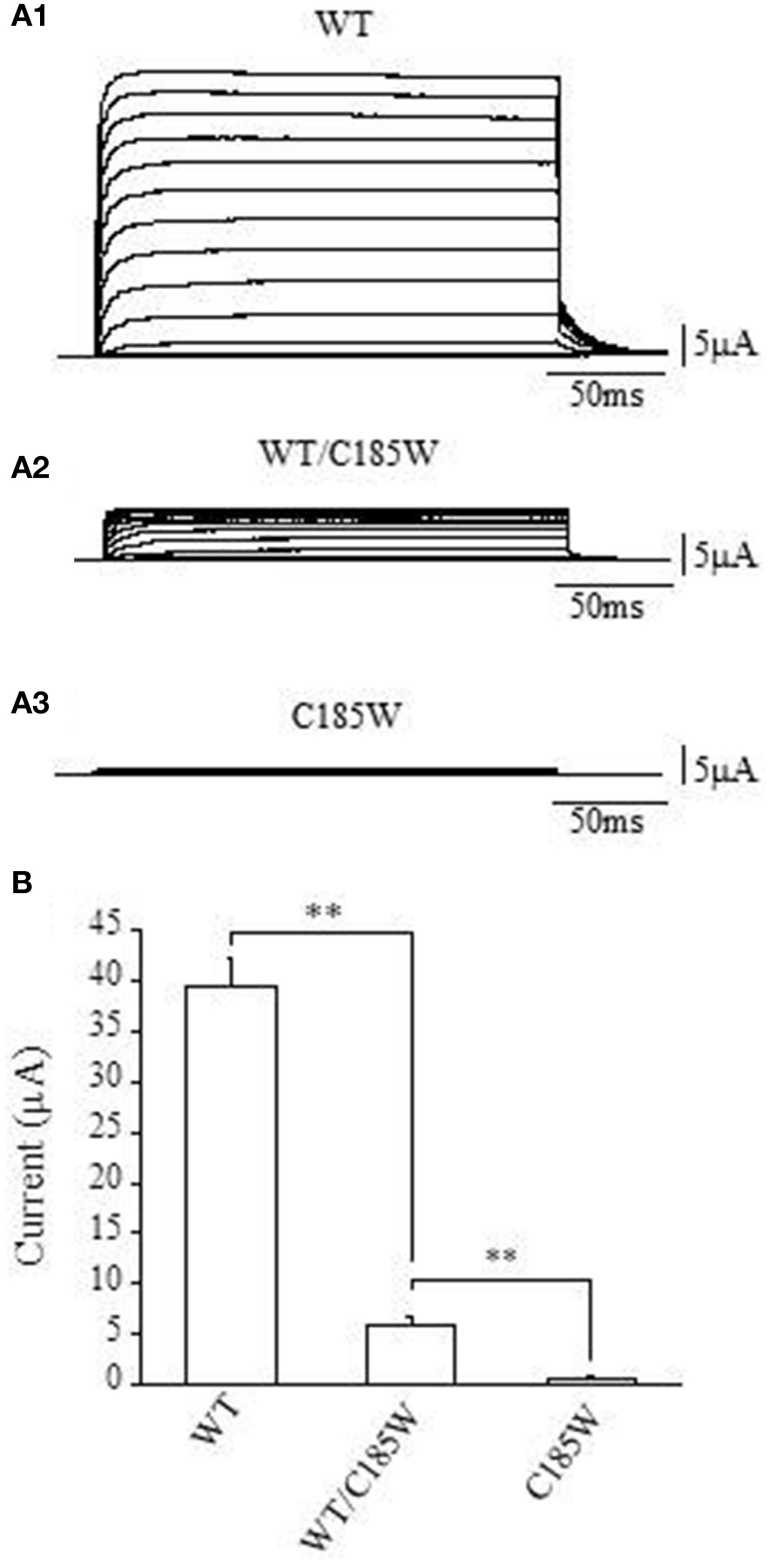
**The C185W mutation results in non-functional homomeric channels and exerts a dominant negative effect on WT channels. (A1–3)** Sample current families recorded from oocytes expressing the indicated channels. Outward currents were evoked by 200 ms depolarizing commands from a holding potential of −80 mV. (**B**) Representative bar graph showing the average whole-cell current recorded at +60 mV from oocytes injected with WT cRNA (2 ng, *left column*), co-injected with WT and C185W cRNAs (1 ng WT + 1 ng C185W, *central column*) or C185W cRNA (2 ng, *right column*). Similar results were obtained from several independent experiments carried out using different batches of oocytes (*not shown*). Data are mean ± SEM of 20–50 cells. The statistical significance was determined by using an unpaired Student's *t*-test (^**^P < 0.001).

*KCNA1* encodes the pore-forming subunit of the voltage-gated Kv1.1 potassium channel. The channel assembles as a tetramer and each subunit contains six transmembrane segments (S1–S6). Both wild-type and C185W mutant channels were expressed in *Xenopus* oocytes and whole-cell currents recorded by TEVC to assess possible differences in the expression levels caused by the mutation. Expression of the homomeric C185W mutant channel resulted in either no measurable functional channel activity, or current amplitudes barely above the background (Figures 2A3, B). By marked contrast, expression of wild-type *Kv1.1* (WT) gave rise to typical delayed-rectifier K^+^ currents of large current amplitudes (Figures 2A_1_, B; cf. Figure 1 in D'Adamo et al., 1998).

The proband was heterozygous for the p.C185W mutation and thus both normal and mutant alleles can be expressed. We therefore examined whether channels composed of a mixture of WT and mutant subunits might be formed. Equal amounts of WT and mutant RNAs were co-injected into the same oocyte and the average current amplitudes then compared with those calculated from cells injected with an equivalent amount of WT RNA. The co-injection of WT and C185W RNAs (1:1 ratio) resulted in a markedly reduced average current amplitude, that was ~13% of the control WT current measured at +60 mV (Figures 2A2, B). Taken together these findings clearly demonstrate that in a homomeric channel the p.C185W mutation causes a near complete loss of function, and also exerts a *dominant negative effect* when combined with WT channels as would be observed in the heterozygous state.

Kv1.1 and Kv1.2 subunits are co-localized in several subcellular brain regions important for the control of movement (e.g., juxtaparanodal regions of myelinated axons and cerebellar Basket cell terminals). These subunits heteromultimerize to form channels whose function are also markedly altered by EA1 mutations (D'Adamo et al., 1999). Consistent with this, we also found that co-expression of Kv1.2 with C185W RNAs (1:1 ratio) resulted in whole-cell current that was ~20% of the control WT, at +60 mV (WT: 27.1 ± 3.2 μA; WT + C185W: 5.4 ± 0.6 μA; C185W: 0.8 ± 0.2 μA; *n* = 6). This demonstrates that the C185W mutation also exerts a dominant negative effect on Kv1.2 channels.

We next focused our investigations on the functional effects of the p.C185W mutation in heteromeric WT/C185W channels. cRNAs for both subunits were co-injected (1:1 ratio) into oocytes. This results in a mixed population of channels where the contribution of non-functional homomeric C185W channels will be negligible whilst functional homomeric WT channels will account for <7% of the channels present. This view is consistent with previous studies demonstrating that EA1 mutations, which do not alter surface expression of the protein (e.g., C185W; Tomlinson et al., 2013), predominantly form heteromeric channels (Imbrici et al., 2011).

**Figure 3:**
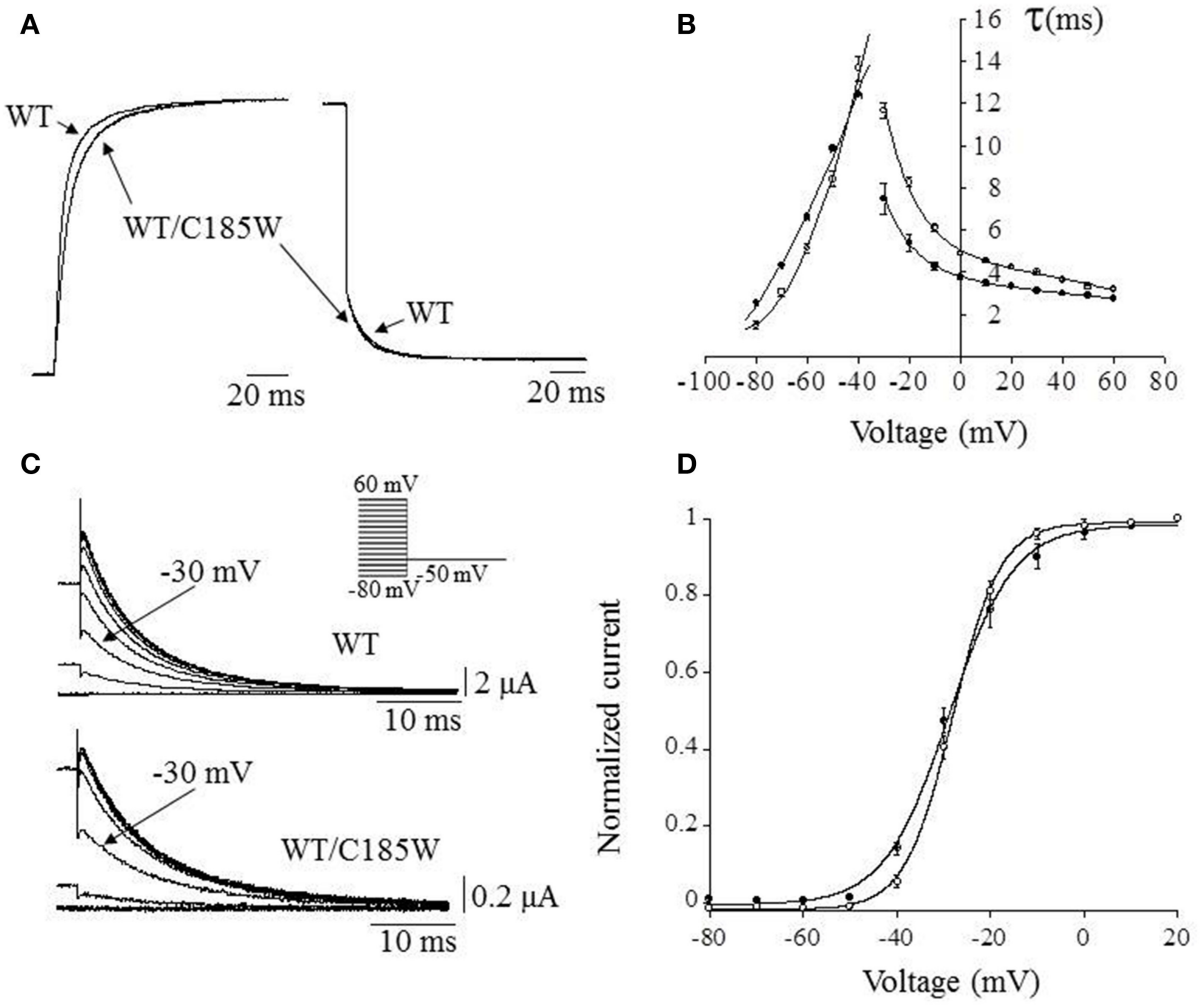
**Effects of the mutation on activation/deactivation kinetics and voltage-dependence of heteromeric channels. (A)** Representative current trace recorded at +60 mV for WT channels, overlain with that resulting from the co-injection of WT + C185W cRNAs (1:1). (**B**) The activating and deactivating current traces were fitted with a double and single exponential function, respectively, in order to assess the effects of the C185W mutation on the activation and deactivation kinetics of the channel. The relevant time constants for the WT (•), WT/C185W (°; 1:1 ratio) channels were calculated and plotted as a function of the test pulse in **(B)**. This plot shows that the mutation affects channel activation (**τ**_fast_) which can be directly observed also from **(A)**. **(C)** Sample tail current families for the indicated channels recorded at −50 mV, after 200 ms pre-pulse commands from −80 mV to 60 mV (*HP* = −80 mV; ΔVincrement = 10 mV, inset). Note that although tail currents for WT/C185W channels are significantly smaller than the WT (*see calibration bars*) the relative current amplitudes, after a pre-pulse potential to −30 mV (*arrows*) are similar, denoting that both channel types have analogous V_1/2_ values. **(D)** The voltage-dependence of channel activation was determined by normalizing tail current-voltage data points for WT (•) or WT/C185W (°; 1:1 ratio) and by calculating the V_1/2_ and slope factor *k* from fits with the Boltzmann function *I* = 1/1 + exp{–(V – V_1/2_)/*k*} (*solid curves*). Notice that the mutation increases the steepness of the voltage-dependence of heterozygous channels.

Whole-cell currents were recorded using tailored voltage-clamp protocols (Figure 3) and the activating/deactivating current traces of either WT or WT/C185W channels were fitted with double- and single-exponential functions, respectively. The calculated time constants were then plotted as a function of membrane potential (Figures 3A,B). This analysis revealed that currents resulting from WT/C185W channels had slower kinetics of activation (Figures 3A,B), whereas the kinetics of deactivation were slightly faster (Figures 3A,B). To determine the voltage-dependence of WT/C185W current activation, tail current families were recorded at −50 mV after pre-pulse commands to several voltages (Figure 3C). The Boltzmann fit to either WT or WT/C185W tail current-voltage data points, revealed that the midpoint activation voltage (V_1/2_) for the WT/C185W current was unchanged (WT: −28.9 ± 0.5 mV; WT/C185W: −28.1 ± 0.5 mV; *p* > 0.05; Figure 3D). However, the slope factor *k* calculated from the Boltzmann fit of tail currents was slightly decreased by the mutation (WT: 6.9 ± 0.3 mV; WT/C185W: 5.1 ± 0.3 mV; *p* < 0.05; Figure 3D).

**Figure 4:**
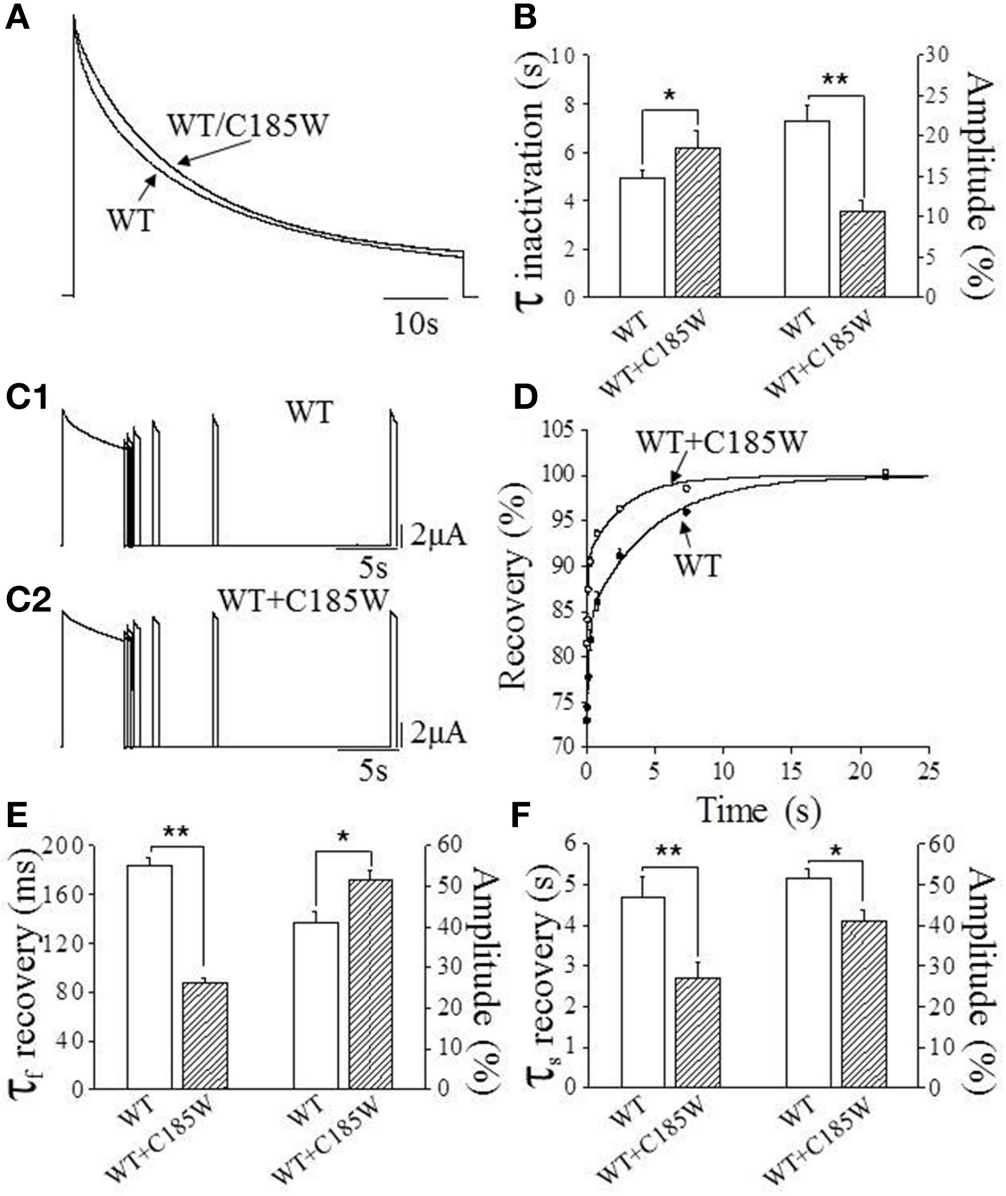
**Effects of the C185W mutation on slow inactivation. (A)** Normalized current traces resulting from either the expression of WT alone or from the co-expression of WT and C185W cRNA at 1:1 ratios. To determine slow inactivation kinetics, a test pulse to +60 mV for 3.5 min was delivered to oocytes expressing WT and WT/C185W channels and the decaying phase of the current fitted with a double-exponential function from which the time constants were calculated. **(B)** Bar graphs showing the fast time constants of the slow inactivation for the indicated channels (*left* y-axis) and the relevant amplitude of time constants (*right* y-axis). These results show that the mutation slightly slows-down the inactivation kinetics of WT/C185W channels. **(C1,C2)** Sample current traces evoked by the two-pulse protocol for the indicated channels. The recovery from slow inactivation was determined for either WT **(C1)** or WT/C185W **(C2)** channels by using a double-pulse protocol to +60 mV, separated by inter-pulse intervals of increasing duration (range: 0.010–21.87 s). The current amplitudes evoked by the second pulse (test) were divided by the first pulse (conditioning), normalized and plotted in **(D)** as a function of the interpulse interval. The solid curves in **(D)** indicate the fit of the data points with a double-exponential function from which the time constants were calculated for WT (•) or WT/C185W (°; 1:1). Bar graphs showing the fast **(E)** and slow **(F)** time constants for the recovery from slow inactivation for the indicated channels (*left* y-axis) and the relevant amplitude of time constants (*right* y-axis). Note that the mutation speeds-up the recovery from inactivation. A careful evaluation of the biophysical properties of homomeric C185W channels was not possible due to marked current reduction. The amplitudes of the time constants were calculated by using the equation: A_fast_ (%) = A_fast_/(A_fast_+A_slow_) × 100. Data are means ± SEM of 10–15 cells. Student's *t*-test: ^*^, *P* < 0.05; ^**^P < 0.001.

WT channels are characterized by a slow process of inactivation named C-type inactivation that increases progressively during intense neuronal activity, modifying both the firing rate and the shape of the action potentials (Aldrich et al., 1979). Thus, possible modifications of this distinct channel property may be of pathogenic relevance for EA1. Slow inactivation was estimated by fitting the time course of current decay with double-exponential functions and calculating the fast (**τ**_fast_) and slow (**τ**_slow_) time constants and relevant amplitudes by using the equation: A_fast_(%) = A_fast_/(A_fast_+A_slow_) × 100. This analysis shows that the **τ**_fast_ for heteromeric WT/C185W channels is slightly slower than the WT (Figures 4A,B). By contrast, the **τ**_slow_ is not statistically different (WT: **τ** = 34.8 ± 2.0 s, amplitude = 64%; WT/C185W: **τ** = 30.6 ± 1.2 s; amplitude = 73%; *p* > 0.05). Remarkably, WT/C185W channels display a much faster recovery from slow inactivation than the WT (Figures 4C–F). These results therefore suggest that this mutation destabilizes the slow inactivated state.

**Figure 5:**
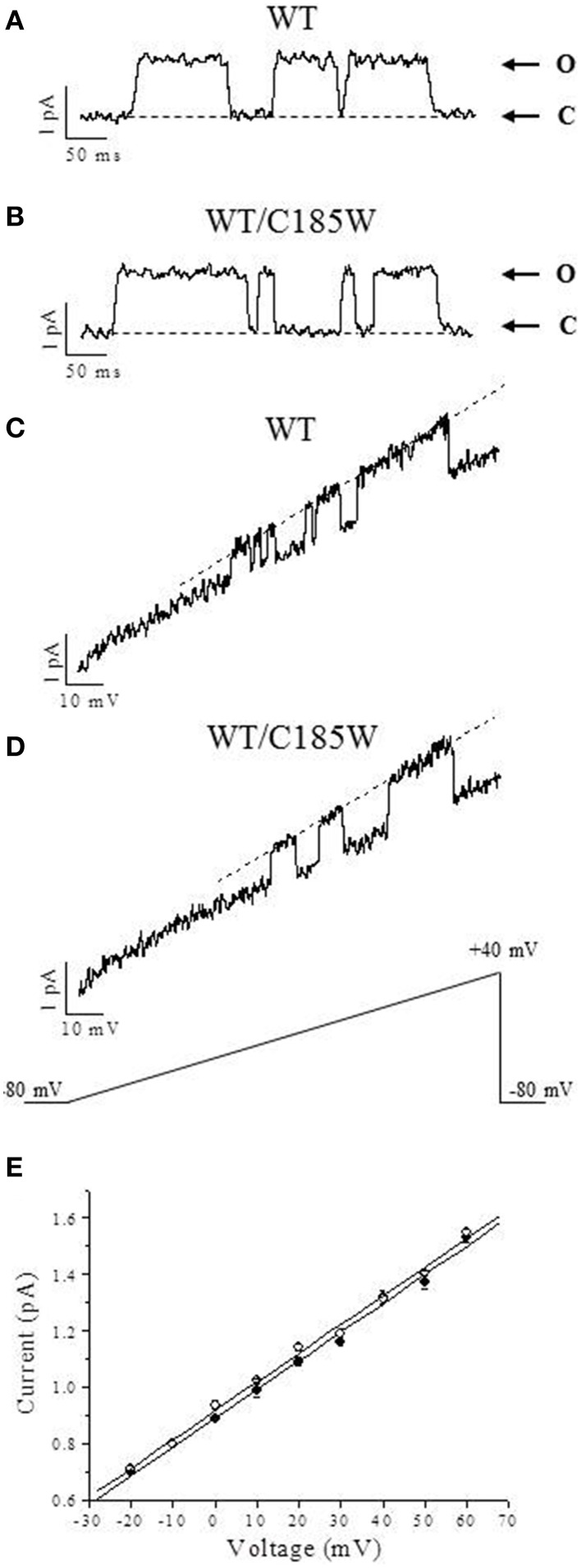
**Analysis of single-channel currents**. Representative single-channel currents recorded in the inside-out configuration of the patch-clamp from oocytes expressing WT **(A)** and WT/C185W **(B)** channels. The openings were evoked by 200 ms depolarizing voltage commands to +20 mV from a holding potential of −80 mV. Channel openings are up-ward deflections and arrows denote closed (C) and open (O) levels. Current traces were filtered at 0.5 kHz. Averaged sweeps with no openings were used to subtract leak and capacity currents. **(C, D)** Single-channel currents recorded from oocytes injected with WT **(C)** or WT/C185W **(D)** cRNAs and evoked by voltage-ramps of 1000 ms duration, from −80 mV to +40 mV. The single-channel current-voltage relationships were calculated from fits of the open state (*dashed lines in C and D*) which yielded 11.4 pS and 10.9 pS for WT and WT/C185W, respectively. **(E)** Single-channel current amplitudes calculated at several voltages from 4 different patches pulled from oocytes injected with WT (•) or WT/C185W (°; 1:1) cRNAs. Note that also these single-channel slope conductance calculations from all event histograms showed no differences between WT (10.8 pS) or WT/C185W (11.2 pS). Data are means ± SEM of 4 patches.

To examine the effects of the p.C185W mutation at the single-channel level, inside-out patch-clamp recordings were performed from oocytes expressing WT or WT/C185W subunits (Figure 5). The single channel slope conductance was unchanged by the mutation (WT: 10.8 ± 0.6 pS; WT/C185W: 11.2 ± 0.5 pS; *n* = 4). Moreover, the open-probability calculated at +20 mV was also unchanged (WT: 0.877 ± 0.022; WT/C185W: 0.882 ± 0.031; *p* = 0.9).

In summary, this detailed functional analysis shows that the C185W mutation: (*i*) causes a nearly complete *loss-of-function* in homomeric channels; (*ii*) exerts a severe dominant-negative effect on WT current amplitudes, and (*iii*) has significant effects on the gating parameters of WT/C185W currents.

### Structural consequences of the p.C185W mutation

**Figure 6:**
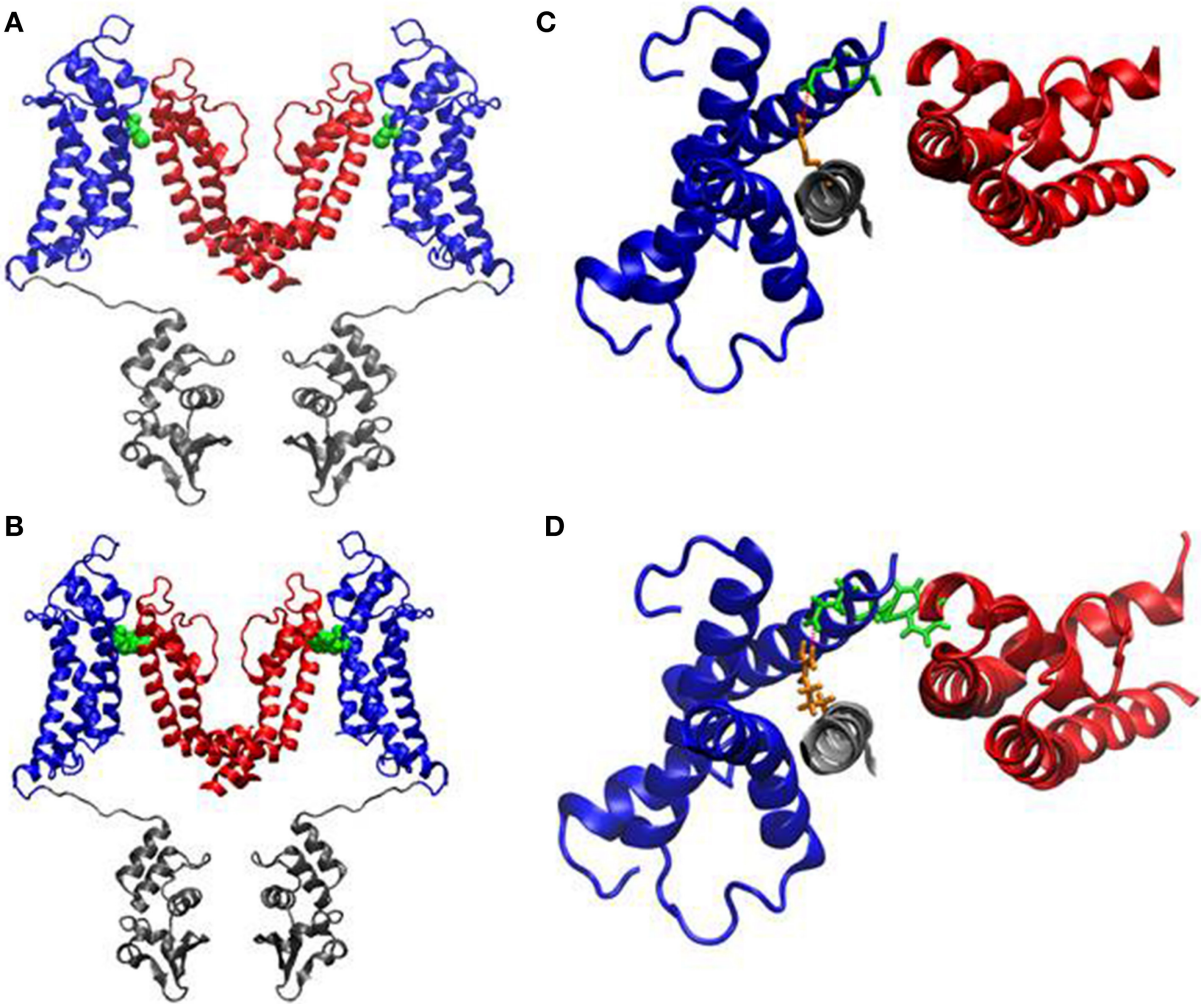
**Homology modeling of the C185W channel. (A, B)** Side stereoviews of a ribbon representation of the pore region (*red*), voltage-sensor (*blue*) and of the T1 domain (*gray*) of the channel. The pictures show only two monomers side by side for clarity. The location of the C185 residue **(A)** and of the C185W mutation **(B)** in the S1 helices are highlighted as *Van der Waals* spheres (*green*). Close-up views of the C185 residue (**C**, *green sticks*) and of the C185W mutation (**D**, *green sticks*) from top, showing their molecular interactions with the P α-helix of the adjacent subunit. The location of the salt-bridge between Glu187 (*green*) and Arg298 (*orange*) is also highlighted as a red dashed line.

To investigate possible structural defects associated with this mutation, we used the crystal structure of the Kv1.2/2.1 channel (Long et al., 2007) to generate a homology model of Kv1.1. Residue C185 in Kv1.1 resides in the S1 segment of the voltage-sensor domain (Figure 6A). Also when mutated *in silico* to a tryptophan the side-chain sterically clashes with the pore-helix domain of the adjacent subunit (Figure 6) and is therefore likely to be highly deleterious to channel function.

## Discussion

Here we describe a single nucleotide mutation that changes a highly conserved residue in the S1 segment (p.C185W) of the Kv1.1 channel subunit in a proband affected by EA1. The mutation produces marked effects on both the channel structure and function, which most likely underscore the several atypical symptoms displayed by the patient. First, we witnessed several episodes of hyperthermia in our proband without an apparent focus of infection. Although spontaneous myokymia was absent in our patient, the episodes of hyperthermia were associated with long lasting attacks of jerking muscle contractions. Thus, a possible explanation for the observed hyperthermia is that such prolonged muscle activity could be responsible for the abnormal rises in body temperature. Exposure to warm temperature has been reported to provoke attacks of ataxia (Eunson et al., 2000; Klein et al., 2004). There are also anecdotal reports of cold temperatures exacerbating symptoms in EA1 patients, which led to the suggestion of temperature extremes triggering EA1 symptoms (Demos et al., 2009). More recently, in Kv1.1 knock-in ataxic mice, spontaneous myokymic activity has been shown to be exacerbated by several types of stimuli including low temperature (Brunetti et al., 2012). Notably, the neuromuscular transmission of EA1 individuals and of animal models of EA1 is temperature sensitive, and attacks of ataxia may be brought on by fever or a hot bath (Tomlinson et al., 2013; D'Adamo et al., 2014). Altogether, this evidence poses the question as to whether loss-of-function mutations in Kv1.1 channels may be responsible for abnormal raises in body temperature by altering the activity of distinct CNS and PNS circuits. Indeed, possible sources of this thermoregulatory defect are hypothalamic neurons that mediate rapid core body temperature changes. Interestingly, these neurons normally express Kv1.1 channels which, if mutated, might produce such effects (Albrecht et al., 1995; Rhodes et al., 1996). In addition, the involvement of distinct subpopulation(s) of peripheral nerves that transmit temperature signals and which also express Kv1.1 channels cannot be excluded (Tsantoulas and McMahon, 2014).

Another atypical symptom, not previously described, concerns the sleeping habits of the patient who reported to need only 5–6 h per day. Interestingly, similar sleeping habits were found in another EA1 patient we have previously investigated (Imbrici et al., 2008). Most people sleep 7–8 h per night, although, a few perform apparently well with just 3–4 h of sleep. This phenotypic trait seems to run in families, although the underlying genes remain unclear. It is interesting to note that mutant *mini-sleep Drosophila*, which sleep ~70% less than WT flies, also carries a loss-of-function point mutation in the *Shaker* (Kv1.1) gene. This mutation also occurs in the S1 domain of the *Drosophila* Kv1.1 ortholog, just three residues beyond the p.C185W mutation (Cirelli et al., 2005). Moreover, mice lacking the *Kcna2* gene, encoding for the *Shaker*-related Kv1.2 channel, also have reduced non-rapid eye movement (NREM) sleep and die early (Douglas et al., 2007). Both Kv1.1 and Kv1.2 can form heteromeric channels in several brain regions, including those involved in sleep-wake rhythm. Moreover, a number of studies have shown that heteromeric Kv1.1/Kv1.2 channels play an important role in the control of neuronal excitability, action potential propagation and synaptic transmission. Previously, we have shown that dominant-negative EA1 mutations can affect the function of heteromeric Kv1.1/Kv1.2 channels (D'Adamo et al., 1999). Here we also demonstrate that C185W markedly reduces the functional activity of Kv1.2 channels. Thus, although polysomnography analysis of the proband was borderline, a role for these channel types in the neuronal processes that control sleep in humans cannot be entirely excluded. Indeed, it is possible that other EA1 patients may display an unrecognized short-sleep duration trait which, if confirmed, could represent the first evidence showing a human K^+^ channel-dependent alteration in sleepbehavior.

Headache is also a symptom over-represented in EA1 individuals. Nevertheless, the presence of long-lasting episodes of severe migraine characterized by nausea, photo- and phonophobia, occurring independently of attacks of EA1, also demonstrate that our probands's symptoms do not fall within the spectrum previously reported for *KCNA1* mutations.

Owing to a noticeable intrafamilial and interfamilial phenotypic variability, genotype-phenotype correlations for EA1 can often be difficult to establish reliably. Indeed, striking differences in severity and frequency of EA1 attacks have been reported even in identical twins (Graves et al., 2010). Thus, the asymptomatic status of the proband's mother and the lack of myokymia in the index patient, although unusual, could be due to low penetrance of the mutation or other epigenetic factors.

### Structural and functional implications

Our structural model shows that the C185W mutation is located very close to a highly conserved salt-bridge (R298/E187 in Kv1.1), occurring between the S1 segment and the S4 helix, which represents the main voltage-sensor of the channel. This interaction, in the Kv1.2/2.1 chimeric channel, has been shown to be important for the control of voltage-dependent gating (Tao et al., 2010). Indeed, these conclusions are also consistent with the tryptophan-substitution mutagenesis of a Kv1.1 channel ortholog (*Shaker*) performed by Hong and Miller, who showed that the C245W mutation (*equivalent to C185W*) alters the voltage-dependence and stabilizes the closed-state of homomeric channels (Hong and Miller, 2000). On the other hand, our structural modeling suggest consistency between the C185W-induced perturbation of the pore-helix and the observed loss-of-function effect, possibly via steric-dependent stabilization of the closed-state in homomeric channel. Severe trafficking defects, underlying the almost complete loss-of-function of homomeric C185W channel, appear unlikely because the mutant protein traffic normally to plasmamembrane when expressed in the human HEK293 cell line (Tomlinson et al., 2013). The structural location of C185W is also coherent with its effects on slow inactivation, a process thought to involve conformational movements of the external pore and selectivity filter (Grissmer and Cahalan, 1989; Stuhmer et al., 1989; Hoshi et al., 1991; Pardo et al., 1992; Lopez-Barneo et al., 1993; Baukrowitz and Yellen, 1995; Molina et al., 1997; Pessia, 2004; González et al., 2012). In particular, the faster recovery from inactivation indicates that the C185W substitution destabilizes the slow inactivated state.

From a neurophysiological perspective, such faster kinetics of recovery from inactivation would render Kv1.1 expressing neurons more readily re-excitable by decreasing the refractory period of action potentials. This would further exacerbate neuronal hyper-excitability induced by the mutation in the heterozygous state.

In conclusion, we report a new EA1 phenotype characterized by hyperthermia associated with long lasting attacks of ataxia and severe migraine. Detailed genetic and functional analysis of this mutant allele are consistent with a pathogenic role for this *KCNA1* mutation. These results therefore indicate that a thorough assessment for hyperthermia, short-sleep trait and migraine should be carefully considered for all patients affected by EA1.

## Author contributions

Mauro Pessia and Thomas Klopstock have been the principal investigators of this study. Thomas Klopstock has been the investigator on industry-sponsored trials funded by Santhera Pharmaceuticals Ltd. (idebenone in LHON, idebenone in Friedreich ataxia) and by H. Lundbeck A/S (carbamylated erythropoietin in Friedreich ataxia); has received research support from government entities (German Research Foundation; German Federal Ministry of Education and Research; European Commission 7th Framework Programme) and from commercial entities (Santhera Pharmaceuticals Ltd.; Actelion Pharmaceuticals Ltd.; H. Lundbeck A/S); has been serving on scientific advisory boards for commercial entities (Santhera Pharmaceuticals Ltd; Actelion Pharmaceuticals Ltd.) and for non-profit entities (Center for Rare Diseases Bonn, Germany; Hoffnungsbaum e.V., Germany); received speaker honoraria and travel costs from commercial entities (Dr. Willmar Schwabe GmbH & Co. KG; Eisai Co., Ltd.; Santhera Pharmaceuticals Ltd; Actelion Pharmaceuticals Ltd; Boehringer Ingelheim Pharma GmbH & Co. KG, GlaxoSmithKline GmbH & Co. KG); has been doing consultancies for Gerson Lehrman Group, USA, and FinTech Global Capital, Japan; has been serving as a Section Editor for BMC Medical Genetics from 2011.

### Conflict of interest statement

The authors declare that the research was conducted in the absence of any commercial or financial relationships that could be construed as a potential conflict of interest.
